# IL-4 and SDF-1 Increase Adipose Tissue-Derived Stromal Cell Ability to Improve Rat Skeletal Muscle Regeneration

**DOI:** 10.3390/ijms21093302

**Published:** 2020-05-07

**Authors:** Małgorzata Zimowska, Karolina Archacka, Edyta Brzoska, Joanna Bem, Areta M. Czerwinska, Iwona Grabowska, Paulina Kasprzycka, Emilia Michalczewska, Igor Stepaniec, Marta Soszynska, Katarzyna Ilach, Wladyslawa Streminska, Maria A. Ciemerych

**Affiliations:** Department of Cytology, Institute of Developmental Biology and Biomedical Sciences, Faculty of Biology, University of Warsaw, Miecznikowa 1, 02-096 Warsaw, Poland; mzimowska@biol.uw.edu.pl (M.Z.); kczaja@biol.uw.edu.pl (K.A.); edbrzoska@biol.uw.edu.pl (E.B.); j.bem@cent.uw.edu.pl (J.B.); aretaczerwinska@biol.uw.edu.pl (A.M.C.); igrabowska@biol.uw.edu.pl (I.G.); p.kasprzycka@biol.uw.edu.pl (P.K.); emiliachudek@student.uw.edu.pl (E.M.); i.stepaniec@gmail.com (I.S.); marta.soszynska@onet.eu (M.S.); kilach@biol.uw.edu.pl (K.I.); krymar@biol.uw.edu.pl (W.S.)

**Keywords:** rat, skeletal muscle, regeneration, ADSC, IL-4, SDF-1

## Abstract

Skeletal muscle regeneration depends on the satellite cells, which, in response to injury, activate, proliferate, and reconstruct damaged tissue. However, under certain conditions, such as large injuries or myopathies, these cells might not sufficiently support repair. Thus, other cell populations, among them adipose tissue-derived stromal cells (ADSCs), are tested as a tool to improve regeneration. Importantly, the pro-regenerative action of such cells could be improved by various factors. In the current study, we tested whether IL-4 and SDF-1 could improve the ability of ADSCs to support the regeneration of rat skeletal muscles. We compared their effect at properly regenerating fast-twitch EDL and poorly regenerating slow-twitch soleus. To this end, ADSCs subjected to IL-4 and SDF-1 were analyzed in vitro and also in vivo after their transplantation into injured muscles. We tested their proliferation rate, migration, expression of stem cell markers and myogenic factors, their ability to fuse with myoblasts, as well as their impact on the mass, structure and function of regenerating muscles. As a result, we showed that cytokine-pretreated ADSCs had a beneficial effect in the regeneration process. Their presence resulted in improved muscle structure and function, as well as decreased fibrosis development and a modulated immune response.

## 1. Introduction

Most of the tissues building mammalian organisms present the capability to self-renew and to regenerate in response to disease or injury. Skeletal muscles are characterized by their exceptional ability to regenerate after physical, chemical, or disease-caused damage. This ability relies on the skeletal muscle stem cells, i.e., satellite cells, which are positioned between the basal lamina and the sarcolemma of skeletal muscle fiber. These unipotent cells remain quiescent until skeletal muscle fiber damage when they resume the cell cycle and form a population of proliferating myoblasts. Next, myoblasts differentiate into myocytes, fuse and form myotubes, which finally mature into functional muscle fibers. Some of the satellite cells do not undergo differentiation, but do restore their own population (for review see [1]). The process of myoblast, myotube, and muscle fiber formation is precisely controlled by the timely expression and action of so-called myogenic regulatory factors (MRFs), such as MyoD, Myf5, and Myogenin [2]. These factors regulate the expression of muscle specific proteins, such as enzymes and elements of unique myofiber cytoskeleton, among them dystrophin and desmin [2,3]. Moreover, the initial stages of satellite cell differentiation occur in the environment of the damaged tissues infiltrated by the immune cells—leukocytes, macrophages, and other cells, such as fibroblasts being involved in the tissue reconstruction [4,5]. All these elements influence skeletal muscle regeneration and impact at the proper or defective outcome of this process. 

Among many factors influencing the progression of regeneration is the type of regenerating muscle—fast-twitch muscles, such as EDL, regenerate properly (e.g., [6,7]). On the other hand, regeneration of slow-twitch muscles, e.g., soleus, is accompanied by the excessive deposition of the connective tissue extracellular matrix (ECM) that leads to the pathological development of fibrosis, severely affecting muscle function. The synthesis of the main components of skeletal muscle ECM, i.e., structural proteins, proteoglycans, and glycosaminoglycans, is precisely regulated during regeneration and is crucial to its outcome (e.g., [8,9]) and the development of fibrosis, i.e., the accumulation of ECM components, such as collagen and fibronectin, leading to the dysfunction of the muscle [10,11]. Thus, successful reconstruction of the extracellular environment of new muscle fibers is one of the most important factors determining the further condition of the tissue and many different strategies are currently used to deal with this issue. Among them are those aiming to modify extracellular environment, for example by inhibiting or activating factors crucial for the regeneration (e.g., [12,13]), or by using various cell populations, as we did in the current study. Nevertheless, the differences between fast- and slow-twitch muscles have to be taken into consideration while assessing the prognosis of the regeneration outcome. These differences rely on the activity of many factors, including the ones involved in the activity of ECM remodeling enzymes, such as matrix metalloproteinases, i.e., MMP-2 and MMP-9, or growth factors, e.g., TGFβ. Thus, interventions that could reduce ECM deposition include inhibition of TGFβ signaling pathways [13,14] or metalloproteinase action [15]. Among the crucial factors underlying these differences is also inflammatory response. Injury causes cells residing within the damaged site, e.g., mast cells, to promote inflammatory reactions. They release pro-inflammatory cytokines, such as IL-6 and IL-8, which attract neutrophils whose initial function is to remove cellular debris [5,8]. Unfortunately, neutrophils can also release cytolytic and cytotoxic molecules that can damage regenerating muscles. Next, pro-inflammatory M1 macrophages (CD68+) enter the site and release pro-inflammatory cytokines, metalloproteases, and many cytotoxic compounds, leading to further muscle damage. M1 are followed by M2 macrophages (CD163+) producing factors that impact satellite cell differentiation and the formation of myotubes and myofibers [4,5,8]. Our previous studies underlined that the differences between fast and slow-twitch muscles in the progression in inflammatory response are closely connected with their ability to effectively regenerate [16].

Except their different abilities to regenerate, such as those observed between slow- and fast-twitch muscles, other circumstances may also affect skeletal muscle repair. Among them are aging, cancer development, or such conditions as myopathies [17]. These can lead to persistent degeneration–regeneration cycles which could result in the depletion of satellite cells preventing muscle reconstruction. Unfortunately, under such circumstances, regeneration improvement could hardly be achieved by the transplantation of satellite cells or myoblasts derived from them. The applicability of these cells in therapies was shown to be limited by their low availability, low survival rate after transplantation, and restricted ability to migrate within the regenerating muscle (e.g., [18,19,20]). For this reason the attention was drawn to other cell types, such as mesoangioblasts [21,22,23], pericytes [24,25], muscle-resident interstitial cells [26], muscle side population cells [27], or muscle-derived stem cells (MDSCs) [28] (for a summary on muscle stem and progenitor cells, see [29]). However, the availability of these cell populations is also limited and for this reason many projects are currently focusing on mesenchymal stromal cells (MSCs), which could be isolated from different niches. Initially, bone marrow was discovered to be the main source of MSCs. However, it has subsequently been shown that MSCs are also present in the blood, umbilical cord, adipose tissue, heart, skin, muscle tissue, liver, gonads, oral and dental tissues, and also other locations. Interestingly, tissue damage or inflammatory response can activate MSCs, as occurs in the case of periapical cysts, which were recently shown to be another promising source of MSCs [30,31]. However, in the case of skeletal muscle therapies, adipose tissue-derived stromal cells (ADSCs), are among the most widely studied MSC populations. MSCs are characterized by the expression of CD105, CD73, and CD90, as well as the absence of CD11b, CD14, CD31, CD34, and CD45 antigens. They are adherent and able to differentiate at least into adipocytes, osteocytes, and chondrocytes [32,33,34,35]. What also has to be taken into account is that MSCs from different sources do not present the same differentiation potential [36]. 

The aim of our study was to characterize the impact of IL-4 or/and SDF-1 on rat adipose tissue-derived stromal cells (rADSCs) at the molecular and cellular level and to compare their fate in two models of rat skeletal muscle regeneration, i.e., (1) well-regenerating fast-twitch EDL and (2) poorly regenerating slow-twitch soleus. Multipotent ADSCs were reported by Gimble and co-workers [37,38,39,40], as well as Zuk and co-workers [41,42,43]. They lack hematopoietic markers such as CD11b and CD45, and express CD13, CD73, CD90, and CD105 [44]. Other markers, such as CD36, could be used to distinguish ADSCs from bone marrow MSCs [45]. It was shown that ADSCs, similarly to MSCs, are able to differentiate into adipocytes, osteocytes, and chondrocytes [46]. Importantly, in vitro differentiation of ADSCs into other cell lineages, such as myoblasts and neurons, was also documented [43]. Many lines of evidence suggest that the paracrine action of MSCs is responsible for their beneficial impact at the tissue regeneration and may be enhanced by the application of selected cytokines or growth factors. Such beneficial action of ADSCs was also documented for skeletal muscles by us [12] and also by others [47,48]. Taking these arguments into consideration, we decided to utilize ADSCs in further analyses. We decided to stimulate them with interleukin-4 (IL-4) and SDF-1, as we previously showed that SDF-1 enhances mouse skeletal muscle regeneration via the stimulation of cell migration and homing [49,50,51,52], while interleukin 4 (IL-4) increases the ability of other stem cell types, i.e., mouse embryonic stem cells, to undergo myogenic differentiation by promoting the expression of early myogenic genes (*Msgn1*, *Pax3*, *Pax7*) [53]. Moreover, IL-4 was shown to be crucial for the proper development, growth and regeneration of skeletal muscle, as it influences the migration and fusion of their cells [54]. Finally, IL-4 also enhances the fusion of stem cells, such as bone marrow-derived MSC or embryonic stem cells with myoblasts [53,55]. The exact mechanism of its action is unknown; however, it was shown, for example, that IL-4 modulates the expression of different cell membrane proteins such as VCAM-1 and integrins [56,57]. Similarly, SDF-1 was also shown to elevate the expression of cell membrane protein—in this case, tetraspanin CD9, which is crucial for the fusion of myogenic cells and can be linked with enhanced mobilization, i.e., the migration and homing of stem cells to the skeletal muscle [58]. 

The impact of ADSCs at the regeneration of rat skeletal muscles was assessed in two diverse and important skeletal muscle models: fast- (EDL) and slow-twitch (soleus) muscles characterized by good and poor regenerative potential, respectively. As mentioned above, the regeneration of EDL and soleus differs at the molecular, cellular, and tissue levels, which we clearly documented in our previous studies [13,15,16,59,60]. Next, we also showed that limiting MMP activity [15] or inflammatory response [16] could improve the repair of poorly regenerating muscles, such as soleus. Thus, in the current study we hoped to uncover whether ADSCs, whose functions we attempted to enhance using IL-4 or/and SDF-1, could be beneficial for the repair of each type of analyzed skeletal muscle. 

## 2. Results

### 2.1. Rat ADSC Response to IL-4 or SDF-1 Treatment In Vitro

**Figure 1:**
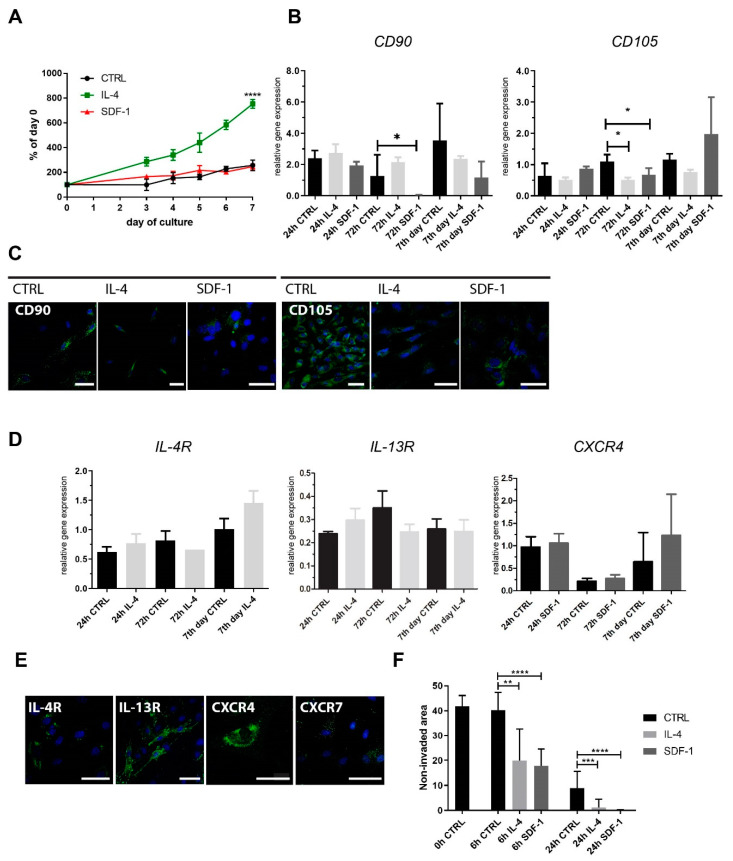
Characterization of rat adipose tissue-derived stromal cells (rADSCs) cultured under control conditions or in the presence of IL-4 or SDF-1. (**A**) Growth curves of rADSCs cultured for 7 days; (**B**) analysis of the level of mRNAs encoding CD90 and CD105. Expression was related to the levels observed in control cells at day 0 (beginning of the culture) and normalized to mRNA encoding HPRT; (**C**) localization of CD90 and CD105 (green) and nuclei (blue) in rADSCs. Bar: 50 µm. (**D**) Analysis of the level of mRNAs encoding IL-4R, IL-13R, and CXCR4. Expression was related to the levels observed in control cells at day 0 (beginning of the culture) and normalized to mRNA encoding HPRT; (**E**) localization of IL-4R, IL-13R, CXCR7, and CXCR4 (green) and nuclei (blue) in control cells. Bar: 50 µm. (**F**) In vitro migration assay—rADSCs were scratched from a culture dish and the area that was not invaded by migrating cells was assessed (6 h and 24 h). N varied between 3 and 9. Data analyzed with Student’s *t*-test: 1A and D (*IL13-R*), data analyzed with Mann–Whitney U test: 1B, 1D (*IL-4R*, *CXCR4*), 1F. Data are presented as mean ± SD. * represents results: * *p* ≤ 0.05; ** *p* ≤ 0.01, *** *p* ≤ 0.001, **** *p* ≤ 0.001. CTRL—non-treated cells.

First, we tested rat adipose tissue-derived stromal cells (rADSCs) that were cultured in vitro in control medium or in the continuous presence of IL-4 or SDF-1. IL-4 significantly increased rADSCs number, which was especially pronounced after 7 days of culture, i.e., we observed approximately four times more cells in the cultures treated with this cytokine compared to control or SDF-1 treated cells (Figure 1A). An analysis of the expression of mRNAs encoding CD90 (Thy-1) and CD105 (endoglin), which are considered the major markers of MSCs [34], showed that neither IL-4 nor SDF-1 influenced *CD90* expression, except for the cells treated with SDF-1 for 72 h. Both treatments led to the downregulation of *CD105* mRNA at 72 h of culture (Figure 1B), which was confirmed by the immunolocalization of this antigen (Figure 1C). Next, we analyzed the expression of IL-4 and SDF-1 receptors and showed that cells expressed mRNA encoding both IL-4 type II receptor subunits, i.e., IL-4R and IL-13R (Figure 1D). 

mRNAs encoding one of the SDF-1 receptors, CXCR4, were expressed (Figure 1D), but CXCR7 was not detectable (data not shown). Moreover, the immunodetection of this antigen did not reveal its presence in rADSCs (Figure 1E). Knowing that SDF-1 and IL-4 could influence cell migration, we performed an in vitro scratch wound healing assay. Rat ADSCs were cultured in control medium or in the presence of IL-4 or SDF-1 until they reached confluency. Next, the scratch was made. The non-invaded area was assessed just after the procedure (0 h) and 6 or 24 h later. Both IL-4 and SDF-1 significantly increased cell migration ability. After 6 h, the non-invaded area was approximately two times smaller in the case of IL-4 or SDF-1 treated cells, and almost completely absent in the case of a longer treatment (24 h) (Figure 1F). 

**Figure 2:**
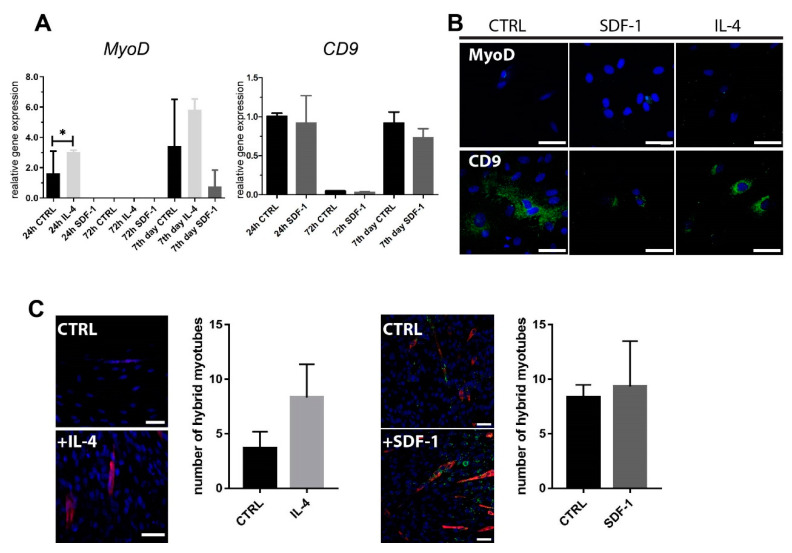
Analysis of expression of mRNA encoding myogenic factors and differentiation potential of rADSCs cultured under control conditions or in the presence of IL-4 or SDF-1. (**A**) Analysis of the level of mRNAs encoding MyoD and CD9. Expression was related to the levels observed in control cells at day 0 (beginning of the culture) and normalized to mRNA encoding HPRT; (**B**) Localization of MyoD and CD9 (green) and nuclei (blue); Bar: 50 µm. (**C**) Co-culture between rADSCs and mouse C2C12 myoblasts. GFP (green), skMyHC (red), nuclei (blue). Bar: 50 µm. N varied between 3 and 6. Data analyzed with of Student’s *t*-test: 2C (IL-4), data analyzed with Mann–Whitney U test: 2A, 2C (SDF-1). Data are presented as mean ± SD. * represents results: * *p* ≤ 0.05. CTRL—non-treated cells.

We assessed how IL-4 or SDF-1 impact the ability of rADSCs to initiate myogenic differentiation in vitro. We evaluated the expression of mRNA encoding mesoderm markers: Brachyury and mesogenin, MRFs Myf5 and MyoD, and adhesion proteins M-cadherin and CD9, but were only able to detect *MyoD*, *CD9*, (Figure 2A) and *Cdh15* (data not shown) mRNAs. Importantly, after 24 h of culture, the level of *MyoD* expression increased significantly in IL-4 treated cells, but it was not sustained in longer culture, nor was it confirmed by immunolocalization (Figure 2A,B). *CD9* mRNA level did not significantly change after SDF-1 treatment (Figure 2A,B). We also did not notice any effect of applied treatment on the ability of BacMam GFP-labeled rADSCs to form hybrid myotubes with mouse C2C12 myoblasts (Figure 2C). Thus, to summarize, in vitro analyses revealed that IL-4 significantly increased the rADSC proliferation rate and that IL-4 and SDF-1 had a significant impact on the cell migration. 

### 2.2. Rat ADSC Impact at the Structure and Molecular Signature of Regenerating Skeletal Muscle 

Our initial analyses showed that rADSCs responded to IL-4 treatment by increasing their proliferation rate (Figure 1A) and to IL-4 and SDF-1 by increasing migration (Figure 1F). Assuming that a combination of these two factors could have beneficial effects on cell proliferation and/or migration, we decided to use both of them for the pre-treatment of rADSC before their transplantation to rat muscles injured by CTX injection. We took advantage of two different muscle models—fast-twitch EDL and slow-twitch soleus—as our previous studies documented the significant differences between them at the molecular, cellular, and tissue level [13,16,59,61]. Most importantly, while EDL regenerates properly, soleus serves as a well-known example of a muscle whose regeneration is affected by the excessive development of connective tissue. 

**Figure 3:**
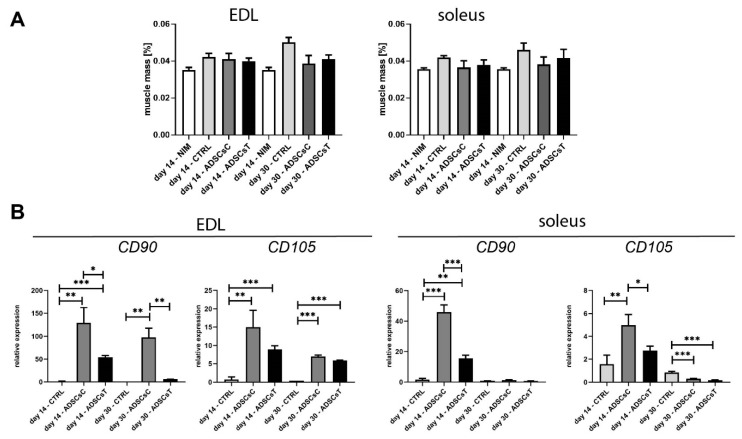
Analysis of muscle mass and stem cell factor expression of rat EDL and soleus skeletal muscles which received control rADSCs or those ones pre-treated with IL-4 and SDF-1. (**A**) Weight of rat skeletal muscles (shown as percent of body mass): non-injured (NIM), injured and injected with NaCl (CTRL), injured and transplanted with control rADSC (ADSCsC) or IL-4 and SDF-1-treated rADSCs (ADSCsT), analyzed at 14 and 30 day of regeneration; (**B**) Expression of mRNAs encoding CD90 and CD105 at 14 and 30 day of regeneration. Expression was related to the levels observed in NIM and normalized to mRNA encoding HPRT. For each experimental group *n* = 3. Data analyzed with of Student’s *t*-test: 3B, data analyzed with Mann–Whitney U test: 3A. Data are presented as mean ± SD. * represents: * *p* ≤ 0.05; ** *p* ≤ 0.01, *** *p* ≤ 0.001.

Control or IL-4 and SDF-1-pretreated rADSCs were transplanted either into EDL or soleus muscles in an autologous manner. An analysis of regenerating muscles isolated at days 14 and 30 after the injury showed no significant influence of rADSC transplantation on muscle weight, presented as a proportion of rat weight (Figure 3A). The presence of rADSCs within the EDL and soleus muscles was indirectly assessed by establishing the levels of mRNAs encoding CD90 and CD105 at days 14 and 30 of regeneration. Control muscles, i.e., those injected with NaCl, did not express significant levels of both transcripts, while they were visibly upregulated in muscles injected with rADSCs (Figure 3B). IL-4 and SDF-1 treatment resulted in the lower expression of *CD90* and *CD105* mRNAs, both in EDL and soleus muscles, compared to the muscles that received control rADSCs (Figure 3B). This was in agreement with our observation showing that, after 72 h of in vitro treatment, both factors downregulated *CD105* expression and SDF-1 also downregulated the expression of *CD90* (Figure 1B). 

**Figure 4:**
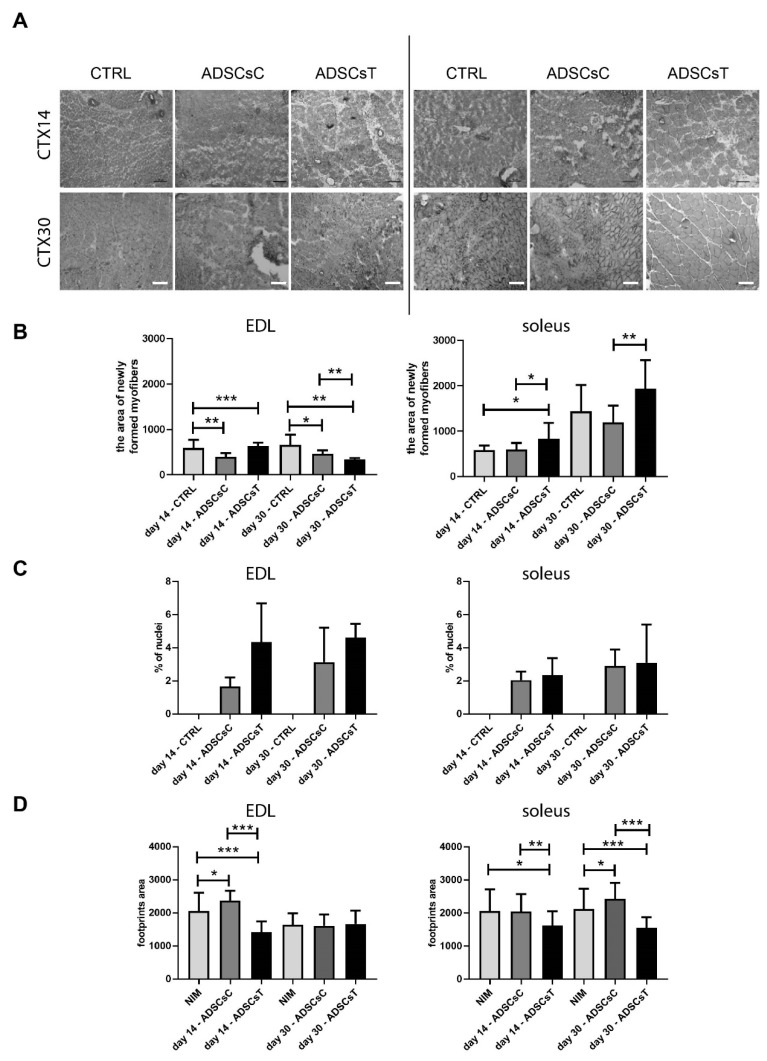
Analysis of muscle morphology, and results of footprint test of rat EDL and soleus skeletal muscles which received control rADSCs or rADSCs pre-treated with IL-4 and SDF-1. (**A**) Morphology of regenerating muscles at days 14 and 30 of regeneration. Hematoxylin/Gomori Trichrom staining; bar: 100 µm. (**B**) Area of newly formed myofibers within the regenerating muscle analyzed at days 14 and 30 of regeneration; (**C**) proportion of centrally positioned nuclei within the regenerating muscle analyzed at days 14 and 30 of regeneration; (**D**) results of footprint test done at days 14 and 30 of regeneration. For 1B n is between 6 and 13, for 1C *n* = 4, for 1D n is between 14 and 39. Data analyzed with of Student’s *t*-test: 4B, data analyzed with Mann–Whitney U test: 4C, D. Data are presented as mean ± SD. * represents: * *p* ≤ 0.05; ** *p* ≤ 0.01, *** *p* ≤ 0.001. Skeletal muscles injured and injected with NaCl (CTRL), injured and transplanted with control rADSCs (ADSCsC) or IL-4 and SDF-1 treated rADSCs (ADSCsT).

The presence of rADSCs resulted in the increased area of newly formed myofibers, i.e., the ones with centrally positioned nuclei (Figure 4C), which were calculated from the analysis of histological sections (Figure 4A). Such an increase was significant in EDL muscles, which received rADSCs pretreated with IL-4 and SDF-1 at day 14 of regeneration (591.9 ± 179.9 and 634.7 ± 71.15, control and ADSCsT, respectively). In the case of poorly regenerating soleus muscles, this effect was noticeable at day 14 and also day 30 of regeneration (day 14—583.2 97.23 and 836.9 ±, 341.5, day 30—1438 ± 538, 1934, ± 629.9 control and ADSCsT, respectively) (Figure 4B). As far as the proportion of centrally positioned nuclei was concerned (Figure 4C), they were visible only in regenerating muscles, however, we did not observe any significant differences between analyzed groups, suggesting that rADSCs pretreated with IL-4 and SDF-1 had a beneficial impact at the diameter of the newly formed myofibers, rather than their number.

Finally, we analyzed how the transplantation of the rADSCs impacted rat walking ability (Figure 4D). In the performed tests, increased footprint area suggests a lack of proper functioning and coordination of tissues in the legs of animals. Our results revealed that the application of rADSCs treated with IL-4 and SDF-1 significantly decreases the impairment caused by the injury, as early as at day 14 of regeneration, regardless of the type of muscle (Figure 4D). Importantly, the reduction in the footprint area was also evident in poorly regenerating soleus muscles injected with IL-4 and SDF-1 treated rADSCs, both at days 14 and 30. Thus, we concluded that transplantation of rADSCs resulted in the improvement of the morphology of regenerating muscles as well as the walking ability of treated animals.

**Figure 5:**
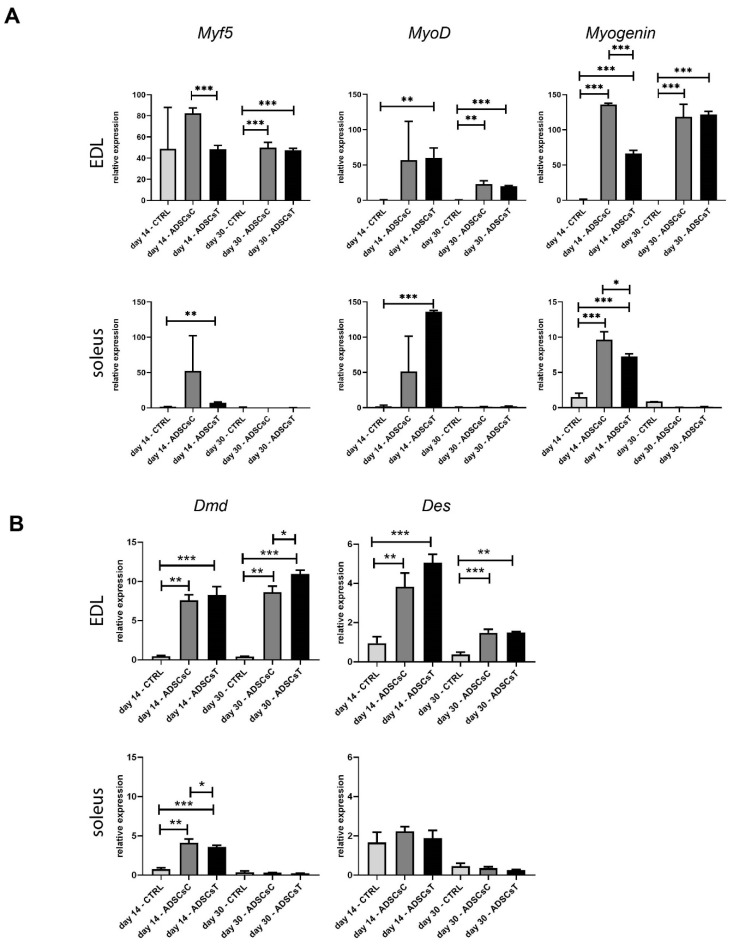
Expression of mRNA encoding myogenic factors in EDL and soleus rat muscles injected with control as well as IL-4 and SDF-1 treated rADSCs. (**A**) Analysis of the level of mRNAs encoding Myf5, MyoD, and Myogenin. Expression was related to the mean expression level observed in control muscle analyzed at day 7 and normalized to mRNA encoding HPRT; (**B**) analysis of the level of mRNAs encoding dystrophin (*Dmd*) and desmin (*Des*). Expression was related to the mean expression level observed in control muscle analyzed at day 7 and normalized to mRNA encoding HPRT. For each experimental group *n* = 3. Data are presented as mean ± SD. * represents results of Student’s *t*-test: * *p* ≤ 0.05; ** *p* ≤ 0.01, *** *p* ≤ 0.001. Skeletal muscles injured and injected with NaCl (CTRL), injured and transplanted with control rADSCs (ADSCsC) or IL-4 and SDF-1 treated rADSCs (ADSCsT).

Simultaneously, we analyzed expression of mRNAs encoding such MRF as Myf5, MyoD, and Myogenin. In the case of EDL, *Myf5* mRNA was expressed at day 14 in all analyzed muscle groups; however, at day 30 it was undetectable in control muscles, which did not receive rADSCs. In soleus muscles, the *Myf5* transcript was present only at day 14. The presence of rADSCs dramatically increased *MyoD* and *Myogenin* expression both in EDL and soleus compared to control muscles. Again, in EDL, these transcripts were detectable at days 14 and 30, while in soleus they were only detectable at day 14. In IL-4 and SDF-1-treated soleus muscles, *MyoD* expression was elevated while *Myogenin* was lower compared to the muscles that received control cells (Figure 5A). At the same time, dystrophin encoding mRNA levels were also higher in muscles receiving rADSCs. The effect of the cells was more pronounced in EDL (days 14 and 30), but was also obvious in soleus (day 14) (Figure 5B). In EDL, the level of desmin encoding mRNA was elevated. Thus, rADSC treatment modified the efficiency of the regeneration process manifested by differences in MRF expression.

### 2.3. Rat ADSCs Pre-treated with IL-4 and SDF-1 Modulate Immune Response in Regenerating Skeletal Muscle 

Two muscles analyzed by us, i.e., EDL and soleus, differ to a great extent in their regenerative potential. These differences also concern the course of inflammatory response that affects the repair efficiency of slow- and fast-twitch muscles. Soleus muscle repair is accompanied by increased and prolonged inflammation compared to EDL. 

**Figure 6:**
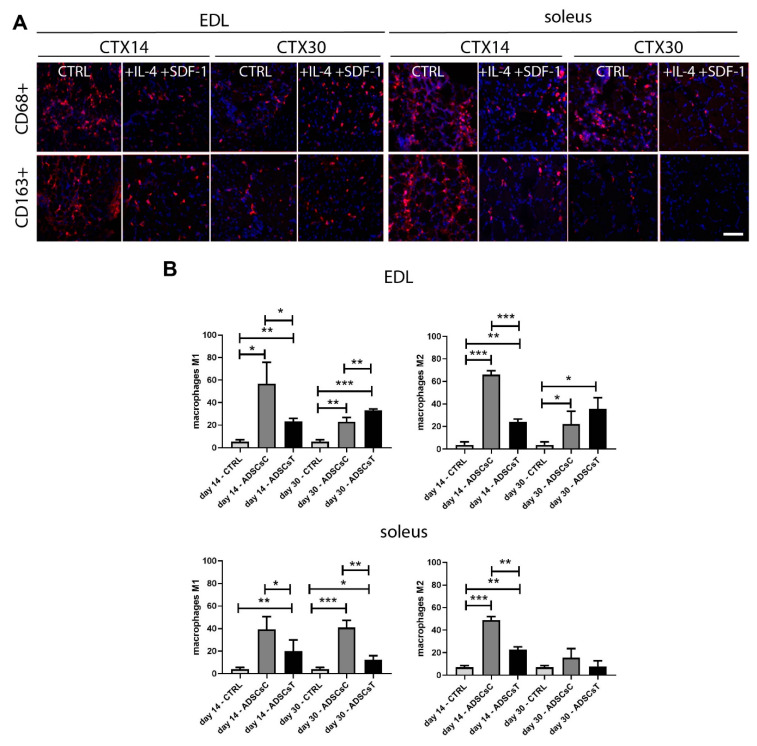
Inflammation development of EDL and soleus rat muscles injected with control (ADSCsC) as well as IL-4 and SDF-1 treated rADSCs (ADSCsT). (**A**) Localization of M1 (CD68+) and M2 (CD163+) macrophages in regenerating muscles at days 14 and 30 of regeneration; bar: 50 µm. (**B**) Number in the field of view of M1 (CD68+) and M2 (CD163+) macrophages in regenerating muscles at 14 and 30 day of regeneration. For each experimental group *n* = 4. Data are presented as mean ± SD. * represents results of Student’s *t*-test: * *p* ≤ 0.05; ** *p* ≤ 0.01, *** *p* ≤ 0.001.

Analysis of the M1 (CD68+) and M2 (CD163+) macrophages in regenerating muscles revealed the impact of ADSCs and IL-4/SDF-1 treatment on inflammatory response during skeletal muscle regeneration. In EDL muscles injected with rADSC, the number of M1 and M2 cells was increased at days 14 and 30 of regeneration. Transplantation of IL-4 and SDF-1 treated rADSCs lowered the number of pro-inflammatory M1 and also anti-inflammatory M2 at day 14 compared to the muscles that received non-treated rADSCs (Figure 6A,B). At day 30, however, the number of both types of M1 and M2 macrophages was higher in muscles transplanted with rADSCs and subjected to IL-4 and SDF-1 treatment (Figure 6B). In soleus, we observed a significantly lower number of M1 in muscles containing rADSCs treated with both factors at both time points. In the case of M2, their number was also lower, but only at day 14 of regeneration (Figure 6B). Thus, rADSCs pretreated with IL-4 and SDF-1 reduced the development of inflammation, especially in soleus muscle, what appears to be crucial for modulating the nature, duration, and intensity of the inflammatory response.

## 3. Discussion

In the current work, we performed in vitro and in vivo analyses, allowing us to characterize rat ADSCs and their responses to two factors known to be involved in myogenic differentiation and cell migration, i.e., IL-4 and SDF-1. IL-4 was previously documented as being secreted by skeletal muscle myoblasts and as having the ability to control the formation of myotubes [54], as well as myogenic precursor cells [57] or epithelial cell migration [62]. It was also shown to upregulate myogenic markers in mouse ES cells [53]. Stromal derived factor-1 (SDF-1, CXCL12) is an effective cell chemoattractant and is also a factor influencing cell survival and differentiation (e.g., [63,64]). Importantly, it was shown to impact at the ability of stem cells to participate in cell fusion, which associates myogenic differentiation, by increasing the expression of CD9 [58]. Thus, by using these two factors, alone or in combination, we attempted to modulate the regeneration-supporting potential of ADSCs and assessed their influence in two models of skeletal muscle repair. 

The regeneration of skeletal muscle is a complex process. Satellite cells, infiltrating inflammatory cells, and the ECM components create a complex signaling environment that contributes to either successful muscle repair or, alternatively, to the development of fibrosis. We analyzed the impact of ADSCs and selected cytokines on EDL, which regenerates properly, and poorly regenerating soleus [6]. In slow-twitch muscles (soleus), the balance between the activation and silencing of individual elements controlling tissue repair, such as ECM remodeling enzymes (MMP-9 and MMP-2), or inflammatory response, is strongly disturbed. As far as inflammation is concerned, muscle damage is associated with the increased production of pro-inflammatory cytokines, such as TNF-α, IL-1, IL-6, and IFN-γ, which are known to have an impact on muscle protein metabolism. It is known that systemic inflammation is associated with reduced rates of protein synthesis and increased protein breakdown, both of which are responsible for the loss of muscle mass. However, the mechanism by which inflammation modulates protein turnover rates is still poorly investigated [65]. In this context, ADSCs appear to play an important role in regeneration. ADSC-treated muscles displayed a reduction in TNF-α, IL-6, and oxidative stress. Interestingly, the level of TGF-β1 was lowered, whereas levels of IL-10 and IL-4 were increased. A decrease in macrophage M1 (CD11 and F4-80) and T lymphocyte (CD3) markers was also observed [66]. Similarly, we observed an increased proportion of M2 macrophages and a higher level of anti-inflammatory factors in muscles, to which Matrigel pretreated with anti-TGF-β1 antibody containing ADSCs was transplanted [12]. Importantly, the beneficial effect of these cells was documented previously in a study focusing on their secretome [47] and one showing that the vascularization of muscles damaged by ischemia could be improved by ADSC transplantation [67]. Thus, we knew that ADSC transplantation could modulate various processes and improve muscle tissue regeneration. 

The differences in the extracellular environment of skeletal muscles can also modulate the behavior of ADCSs transplanted to them and, consequently, the effectiveness of the regeneration. Moreover, pre-treatment with IL-4 and SDF-1 modifies the features of ADSCs. Our in vitro studies showed that the presence of IL-4 significantly increased rADSC proliferation. The migration ability of these cells was improved by both IL-4 and SDF-1, but none of these factors increased the ADSCs’ potential to fuse with mouse C2C12 myoblasts. It was previously shown that MSCs from bone marrow and Wharton’s jelly are able to interact with myoblasts and form hybrid myotubes and that SDF-1 or IL-4 improve this process [50,51,55,68]. We also showed that human cells, e.g., those isolated from connective tissue of umbilical cord or bone marrow-derived mesenchymal cells, can form hybrid myotubes with mouse myoblasts [51,68]. Interestingly, SDF-1 which was shown by us to increase CD9 expression in mouse MSCs [50,58], did not have such an impact on rat ADSCs. 

Our observation supports the notion that the myogenic differentiation of MSCs, including ADSCs, is still a challenging task. Many reports show that MSC multipotency can be manifested also by their ability to form myoblasts [69]. However, MSC differentiation is still inefficient. As we previously showed, such cells were able to form hybrid myotubes with C2C12 cells in vitro and to colonize skeletal muscle tissue and improve its regeneration [51]. Furthermore, others documented that MSCs (e.g., [70,71,72]) and also ADSCs [48] were able to participate in new myofiber formation, albeit with very low frequency. Importantly, even if MSCs did not participate in the formation of new myofibers, their presence was beneficial for regeneration due to their immunomodulatory function, as shown by us [12] and also others (e.g., [73,74,75]). Moreover, it is possible that certain subpopulations of ADSCs are characterized by their varying potential to differentiate, which might translate to their ability to support tissue healing [76,77]. 

Our current in vivo analyses proved that ADSCs were able to improve skeletal muscle regeneration. It was particularly evident in poorly regenerating slow-twitch soleus muscles: the injection of injured muscle with either control ADSCs or that pre-treated with IL-4 and SDF-1 improved its morphology and led to the formation of larger and more homogeneously sized fibers compared to those observed in control muscles. The development of fibrosis, characteristic of poorly regenerating soleus muscles, was also limited and the area of newly formed myofibers increased. Next, ADSC transplantation increased MRF expression at days 14 and 30 in EDL and only at day 14 in soleus muscle. Interestingly, we did not detect any striking differences in MRF levels between the EDL muscles that received control and cytokine-treated ADSCs. Importantly, however, in regenerating soleus muscle, IL-4 and SDF-1 upregulated *Myf5*, *MyoD* and *Myogenin* expression compared to control muscles, but only at day 14 of regeneration. It seems that the major mode of ADSC action was attributed to their immunomodulatory ability, which could be enhanced by IL-4 and SDF-1. In soleus muscle, transplantation of ADSCs pre-treated with IL-4 and SDF-1 reduced number of M1 and M2 macrophages during muscle regeneration, whereas in EDL muscle this reduction was visible only at day 14 of tissue repair. However, it must be remembered that, in EDL, the repair process is more rapid compared to soleus and, at day 14 after the damage, EDL is already substantially rebuilt.

## 4. Materials and Methods 

All procedures involving animals were approved by Local Ethics Committee No. 1 in Warsaw, Poland—permission number: 493/2018 (January 2018). At least three independent experiments were performed for each analysis presented and a detailed N number was indicated in each figure legend. 

### 4.1. Cell Isolation and Culture

Rat adipose tissue was isolated from Wistar rats (age 8–10 weeks), transferred to betadine solution (8 µL/mL, EGIS Polska sp. z o.o.) in phosphate buffer saline (PBS) and then washed with PBS. Rat adipose tissue-derived stem/stromal cells (rADSCs) were isolated by the digestion of fragmented adipose tissue with 0.2% type I collagenase solution (Sigma-Aldrich) for 90 min at 37 °C. Cells were cultured in high glucose Dulbecco’s Modified Eagle’s Medium (DMEM, Invitrogen) supplemented with 10% of fetal bovine serum (FBS, Invitrogen) and 10 μg/mL gentamycin (Sigma-Aldrich) in 5% CO_2_ at 37 °C. The culture medium was replaced every 2–3 days and cells were passaged when they reached confluency. For further experiments, cells from passages three to seven were used. 

### 4.2. Cell Treatment with IL-4, SDF-1, and IL-4 and SDF-1

Rat ADSCs were cultured at 5 × 10^3^/cm^2^ density in high glucose DMEM supplemented with 10% FBS and 10 μg/mL gentamycin in the absence or presence of either 10 ng/mL of IL-4 (recombinant rat IL-4 504-RL R&D Systems) or 25 ng/mL of SDF-1 (recombinant rat SDF1 protein ab243782 Abcam) for 7 days for cell proliferation and migration, qPCR and immunolocalization analyses. The culture medium was replaced every day to keep the required concentration of the abovementioned factors. The morphology of cells was analyzed using a Nikon Eclipse TE200 microscope with a Hoffman contrast. Cell proliferation was assessed for control and treated rat ADSCs every day from days 3 to 7 of the culture. For qPCR and the immunolocalization analyses of the control, IL-4 treated and SDF-1 treated rADSCs were collected after 24 h, 72 h, and 7 days of the culture. 

### 4.3. Migration Assay

The migration of ADSCs was analyzed using scratch wound healing assay [58]. Briefly, rADSCs were cultured in high-glucose DMEM supplemented with 10% FBS and 10 μg/mL gentamycin in the absence or presence of either 10 ng/mL of IL-4 or 25 ng/mL SDF-1 until 90% of confluency. Next, the cells were scratched from the plate using a plastic automatic pipette tip to create the “wound”. The wound healing manifested by the ability of cells to refill the created gap was monitored after 6 h and 24 h of the culture. 

### 4.4. Co-culture of ADSCs with C2C12 Myoblasts

For co-culture experiments, 25 × 10^3^ mouse C2C12 myoblasts (Sigma–Aldrich, St. Louis, MO, USA) and 25 × 10^3^ of rADSCs were seeded on sterile coverslips and cultured in high-glucose DMEM supplemented with 10% of FBS and 10 μg/mL gentamycin, in the presence or absence of IL-4 or SDF-1 for 7 days. Rat ADSCs used for the co-culture were first labelled with BacMam GFP Transduction Control (Invitrogen), according to the manufacturer’s instructions. Culture medium was replaced every day to keep required levels of cytokines. Cells were fixed with 3% paraformaldehyde (PFA) and subjected to immunolocalizatio analysis. BacMam GFP allowed for the identification of hybrid myotubes, i.e., ones generated by rADSCs fusing with C2C12 myoblasts. Nuclei were stained with Hoechst 33,342 (Sigma Aldrich) or DRAQ5 (Biostatus Limited), diluted 1:1000 in PBS. In selected experiments, cells were stained with either anti-myosin (skeletal) antibody (M7523, Sigma Aldrich) or anti-skeletal muscle marker (12/101-c, DSHB) to visualize the localization of myotubes in the culture. For each experimental group, the number of hybrid myotubes was counted from at least five fields of view of three independent experiments. 

### 4.5. Preparation of Cells for Transplantation 

Rat ADSCs, labelled with BacMam GFP Transduction Control (Invitrogen), were cultured in high-glucose DMEM supplemented with 10% FBS and 10 μg/mL gentamycin in the absence or presence of 10 ng/mL of IL-4 and 25 ng/mL of SDF-1 for 48 h. Next, cells were washed with PBS and detached with 0.05% trypsin (Invitrogen). Cells were collected, centrifuged and suspended in 0.9% NaCl solution at 5 × 10^5^ cells/mL density for transplantation studies.

### 4.6. Skeletal Muscle Injury

After the anesthesia of the rats (8–10 week old male Wistar) with isoflurane, EDL and soleus muscles were exposed and damaged by 25 µL of 100 μM cardiotoxin (Latoxan) injection. The surgical field was also locally anesthetized with 2% Lidocaine. Next, injured muscles were injected with 40 µL of 0.9% NaCl solution containing either control (i.e., untreated) or treated rADSCs, as described above. In control experiments, injured muscles were injected with 0.9% NaCl solution only or muscles were not treated, i.e., they remained intact (hereafter referred to as non-injured/injected muscle (NIM)). Each variant of the experiment was performed with at least three biological repeats. After the surgery, the animals were kept under standard conditions with free access to food and water. They received liquid paracetamol (in drinking water) for 2 days (100 mg/kg of body weight/day). 

### 4.7. Footprint Area Measurements

Footprint test was conducted with rats, controls and those whose muscles received ADSCs, at days 14 or 30 of regeneration. Hind limbs were painted with ink and animals were then allowed to walk along a runway, leaving their footprints on paper. Footprint area was measured in ImageJ 2.0 and was shown as the ratio of the footprint area made by the leg with regenerating muscles to that of legs whose muscles were uninjured. 

### 4.8. Histological Analyses—Myofiber Number and Connective Tissue Area

Rats were euthanized by CO_2_ exposure. Muscles, and animals from which the muscles were taken, were weighed. The isolated muscles were frozen in isopentane, cooled in liquid nitrogen and stored at −80 °C. The frozen rat muscles were cut into sections of 10 μm thickness using cryostat (Microm HM505N). Cross-sections were placed on slides and, after drying, were stored at 4 °C. Sections were hydrated in PBS (10 min), and then stained with Hematoxylin and Gomori Trichrom (Merck), according to the manufacturer’s instructions. The stained sections from each muscle were analyzed using a Nikon TE200 microscope and the NIS Elements program. Next, the mean area of 100 regenerating myofibers, as well as the area occupied by the connective tissue in relation to the area of the entire section, were both determined using ImageJ 2.0 software for each muscle. 

### 4.9. qPCR

RNA was isolated from rADSCs using a High Pure RNA Isolation Kit (Roche) and from muscles using an mirVana PARIS Kit (Thermo Fisher Scientific), according to the manufacturer’s instructions. Reverse transcription was performed using 0.5 μg total RNA and a RevertAid First Strand cDNA Synthesis Kit (Thermo Fisher Scientific), according to the manufacturer’s instructions. qPCR was performed using specific TaqMan^®^ probes (listed in Supplementary Table S1), TaqMan Gene Expression Master Mix (Thermo Fisher Scientific) and Light Cycler 96 instrument (Roche). Data were collected and analyzed with Light Cycler 96 SW1.1 software (Roche). An analysis of relative gene expression was performed according to [78]. 

### 4.10. Immunolocalization 

Control and IL-4 or/and SDF-1 treated rADSCs were fixed with 4% paraformaldehyde (PFA, Sigma-Aldrich) for 15 min after 1, 3 and 7 days of the culture. Cells were washed in PBS (10 min), incubated in 0.2% Triton-X100 (Sigma-Aldrich) in PBS (5 min), 0.25% glycine (Sigma-Aldrich) in PBS (30 min), 3% BSA in PBS (60 min), and finally in selected primary antibody solution in 3% BSA in PBS (2 h). The following primary antibodies were used: anti-CD105 (mouse monoclonal ab11414, Abcam); anti-CD90 (mouse monoclonal ab225, Abcam); anti-CXCR4 (rabbit monoclonal ab124824, Abcam); anti-CXCR7 (rabbit polyclonal ab117836, Abcam); anti-IL4R (rabbit polyclonal NBP1-00884, Novus Biologicals); anti-IL13R (rabbit polyclonal NBP1-61690, Novus Biologicals); anti-MyoD (rabbit polyclonal c-20, Santa Cruz Biotechnology) and anti-CD9 (rabbit monoclonal ab92726, Abcam). After PBS washing, cells were incubated with appropriate secondary antibodies, diluted 1:200 in 3% bovine serum albumin (BSA, Sigma-Aldrich) in PBS (2 h), and then in Hoechst or DRAQ5, diluted 1:1000 in PBS (5 min). Muscle cross-sections were rehydrated for 10 min in PBS, and then fixed for 10 min in 3% PFA in PBS. Next, sections were permeabilized with 0.1% Triton X-100 in PBS, and incubated with 0.25% glycine for 15 min. The non-specific binding of antibodies was blocked with 3% BSA in PBS, at room temperature, for 30 min. Next, sections were incubated with selected primary antibodies, diluted 1:100 in 3% BSA in PBS, overnight. The following primary antibodies were used: anti-CD68 (rabbit monoclonal ab53444, Abcam), anti-CD163 (rabbit monoclonal ab182422, Abcam) and anti-GFP (rabbit polyclonal ab6556, Abcam). After PBS washing, sections were incubated at room temperature with appropriate secondary antibodies, diluted 1:200 in 3% BSA in PBS. Cell nuclei were visualized by incubation with DRAQ5, diluted 1:1000 in PBS, for 10 min. All specimens were mounted with Fluorescent Mounting Medium (Dako Cytomation) and analyzed using an Axio Observer Z1 LSM 700 scanning confocal microscope (Zeiss) equipped with Zen software (Zeiss). 

### 4.11. Statistical Analysis

Results were analyzed using GraphPad Software (San Diego, CA, USA). All experimental groups were analyzed using a Shapiro–Wilk normality test. A non-paired Student’s *t*-test was performed for the data that passed the normality test, a Mann–Whitney U test was performed for data whose distribution was abnormal. Data are presented as mean ± standard deviation. Statistical significance was determined using a Student’s *t*-test or a Mann–Whitney U test (* *p* < 0.05; ** *p* < 0.01; *** *p* < 0.005, **** *p* < 0.0005).

## 5. Conclusions

Our study shows that IL-4 and SDF-1 modulate the action of rADSCs. IL-4 increased rADSC proliferation, while combined treatment with IL-4 and SDF-1 significantly improved the muscle regeneration-supportive action of these cells. Importantly, the use of rADSCs treated with both cytokines improved the repair of poorly regenerating slow-twitch soleus rat muscles. Thus, the application of ADSCs in combination with properly selected cytokines could be considered as a tool to modulate skeletal muscle regeneration.

## Supplementary Materials

Supplementary Materials can be found at https://www.mdpi.com/1422-0067/21/9/3302/s1.

## Abbreviations

ADSC	adipose tissue-derived stromal cell
ECM	extracellular matrix
IL-4	interleukin-4
TGFβ	transforming growth factor beta
SDF-1	stromal cell-derived factor-1
