# Supplementary material for: IL-4 and SDF-1 Increase Adipose Tissue-Derived Stromal Cell Ability to Improve Rat Skeletal Muscle Regeneration
Source: Int J Mol Sci. 2020 May 7;21(9):3302. doi: 10.3390/ijms21093302 (PMC7246596; doi:10.3390/ijms21093302)
Supplement: Supplementary file 1 [file ijms-21-03302-s001.zip › ijms-758744-SI.pdf]

Supplementary Table S1. Taqman assays used in qPCR analyzes.

**Gene**	**Taqman assay no.**
CD90	Rn00562048_m1
CD105	Rn01438763_m1
IL4Rα	Rn01507024_m1
IL13Rα1	Rn01457337_m1
CXCR4	Rn01483207_m1
Brachyury	Rn01527349_m1
Cdh15	Rn01432568_m1
MyoD	Rn01457527_g1
Myf5	Rn01502778_m1
Myog	Rn00567418_m1
CD9	Rn01463253_m1
HPRT	Rn01527840_m1
Des	Rn00574732_m1
DmD	Rn00562253_m1
Msgn1	Rn01473566_s1
